# Exploring Subtilisin Inhibition to Discover Antimalarial Drugs: Insights into Medicinal Chemistry and Drug Discovery

**DOI:** 10.3390/ph18091318

**Published:** 2025-09-03

**Authors:** Margarida Cochicho Leonardo, Sonaly Lima Albino, Wallyson Junio Santos de Araújo, Maria Verônica de Barros Nascimento, Juan David Rodríguez-Macías, Edgar Alexander Marquez Brazon, Ricardo Olimpio de Moura, Fátima Nogueira, Igor José dos Santos Nascimento

**Affiliations:** 1 Global Health and Tropical Medicine (GHTM), Associate Laboratory in Translation and Innovation Towards Global Health (LA-REAL), Instituto de Higiene e Medicina Tropical (IHMT), Universidade NOVA de Lisboa (UNL), Rua da Junqueira 100, 1349-008 Lisbon, Portugal; mc.leonardo@campus.fct.unl.pt (M.C.L.); fnogueira@ihmt.unl.pt (F.N.); 2 Postgraduate Program of Pharmaceutical Sciences, Department of Pharmacy, State University of Paraíba, Campina Grande 58429-500, PB, Brazil; sonaly.albino@hotmail.com (S.L.A.); ricardo.olimpiodemoura@servidor.uepb.edu.br (R.O.d.M.); 3 Drug Development and Synthesis Laboratory, Department of Pharmacy, State University of Paraíba, Campina Grande 58429-500, PB, Brazil; wallyson.araujo@aluno.uepb.edu.br (W.J.S.d.A.); maria.veronica@aluno.uepb.edu.br (M.V.d.B.N.); 4 Facultad de Ciencias de la Salud, Exactas y Naturales, Universidad Libre Seccional Barranquilla, Barranquilla 080001, Colombia; juand.rodriguezm@unilibre.edu.co; 5 Facultad de Ciencias Básicas, Departamento de Química y Biología, Universidad del Norte, Barranquilla 081007, Colombia; ebrazon@uninorte.edu.co

**Keywords:** *Plasmodium*, antimalarial drugs, subtilisin, SUB1, SUB2, SUB3

## Abstract

**Introduction:** Malaria is a tropical disease caused by the parasite *Plasmodium* sp., which is considered a significant public health challenge, particularly in Africa. Among the species related to human infection, *P. falciparum* and *P. vivax* are known for their high incidence and pathogenicity. Despite several approved drugs in the treatment, the increase in resistance mechanisms is becoming increasingly prevalent, which makes the discovery of effective and safer drugs challenging. Thus, it is necessary to explore new mechanisms of action for the discovery of innovative antimalarial agents. Among the explored targets, proteases, especially subtilisin, have shown great promise in the development of new therapeutic options. **Method**: A narrative review was conducted using the main databases to provide critical information about the subtilisin to design antimalarial drugs. **Results:** Critical data were found about the isoforms of subtilisins, highlighting SUB1 and SUB2. SBDD approaches were able to show that compounds designed to target the catalytic Asp^372^, His^428^, and Ser^606^, and other such Leu^469^, Gly^467^, and Asn^520^ against SUB1, presented critical results. In addition, quinoline, benzopyran, and triterpene derivatives and peptide inhibitors show their importance, and these scaffolds can be explored in further work. **Conclusions**: Considering the relevance of this target, this review provided insights into medicinal chemistry, the discovery of antimalarial drugs that act by inhibiting subtilisin, and promoted a promising initiative to combat malaria.

## 1. Introduction

Malaria is one of the most impactful infectious diseases in tropical and subtropical regions and is caused by *Plasmodium* sp. and transmitted through the bite of infected female *Anopheles* mosquitoes [1,2]. Among the species, those that commonly parasitize humans are caused by *P. falciparum* and *P. vivax*, the most prevalent and severe forms of the disease [3]. According to the World Health Organization (WHO), in 2023, approximately 263 million cases were reported, with Africa being the most affected region, accounting for 4% of the global cases [4]. The occurrence of this disease is influenced by economic, environmental, and sociocultural factors, prompting researchers worldwide to develop new drugs against it [5].

One of the primary challenges in controlling malaria is the development of resistance to antimalarial drugs, combined with insecticide resistance in mosquito vectors and the lack of an efficacious vaccine [6]. These factors make treatment difficult by limiting access to drugs with better safety and efficacy profiles [7]. The continuous emergence and spread of *Plasmodium falciparum* resistance to antimalarials, which have been successively developed and introduced into clinical practice, pose a significant threat to global malaria control and eradication efforts. Therefore, there is an urgent need to develop new therapeutic agents that can overcome resistance mechanisms by interacting with essential targets in the parasite’s life cycle [8]. In addition, exploring new mechanisms of action can contribute to drug innovation and help overcome the current mechanisms of resistance that limit the effectiveness of actual therapies [9,10]. Thus, among the explored targets, proteases can yield significant results and contribute to the development of a critical antimalarial drug [11,12].

Proteases are a family of enzymes that play a crucial role in the parasite’s life cycle, making them a primary target for the development of new antimalarial chemotherapy strategies [13]. Thus, cysteine and serine proteases can be cited as potential targets for the design and discovery of antimalarial agents. Cysteine protease is an enzyme involved in host–parasite interactions mediating crucial metabolic processes, and serine protease is related to parasite replication and survival, in addition to acting in disease progression through protein activation [14,15]. In this way, several studies highlight the importance of these structures in drug design for discovering new drugs focused on malaria [16,17].

Among the serine proteases, subtilisin plays a crucial role in the life cycle of malaria, especially in the asexual blood stages of *P. falciparum*, which are responsible for the clinical manifestations of the disease [11]. This enzyme acts on the release of merozoites from infected erythrocytes and on the invasion of new blood cells. Thus, studies involving subtilisin inhibition represent a promising field for the discovery of drugs based on inhibitors of parasite proliferation in the bloodstream, reducing disease progression. Additionally, exploring this target can provide a new class of antimalarials that can overcome the parasite’s resistance mechanisms to existing drugs [18].

Furthermore, medicinal chemistry plays a crucial role in the optimization and discovery of bioactive substances of therapeutic interest, primarily for the treatment of malaria [19,20]. Over the years, the methods have evolved, making computer-aided drug design (CADD) a widely used tool in medicinal chemistry research. This approach promotes agility in the drug development process, allowing for faster and lower-cost identification of new drugs and reducing failures during this process [19,21,22]. One of the most prominent approaches in CADD is structure-based drug design (SBDD), which aims to improve the affinity and specificity of drug-receptor interactions, yielding promising results in the discovery of new antimalarial drugs targeting subtilisin [23,24,25,26].

Ultimately, this review aims to discuss potential antimalarial drugs based on subtilisin inhibition, with the goal of providing new insights that can be applied to develop safer, more effective, and less toxic drugs using medicinal chemistry strategies. Our search was conducted in the ScienceDirect, PubMed, and Google Scholar databases using the descriptors “subtilisin” and “antimalarial drugs”. Articles related to the topic were selected without a time limit. Furthermore, we aim to demonstrate the importance of in silico and in vitro studies in the rational design of drugs, enabling the discovery of more promising and efficient compounds for malaria treatment.

## 2. Malaria: An Overview

Between 1970 and 2000, a global resurgence of malaria occurred, driven by the increasing resistance of *Anopheles* spp. mosquito vectors to insecticides and of *Plasmodium falciparum* parasites to commonly used antimalarial drugs. This trend was subsequently reversed between 2000 and 2015, during which time the global malaria burden declined significantly, and malaria-related mortality was reduced by more than half [27,28]. However, since 2015, estimated malaria cases in sub-Saharan Africa have steadily increased, a trend that has been further aggravated since 2020 by the health system disruptions associated with the COVID-19 pandemic [29,30]. It is estimated that malaria caused more than 260 million infections in 2023 (11 million more than in 2022) across the 83 endemic countries, with a death toll of more than half a million people, highlighting the impact of the disease on global human health [31]. Of the five species that cause malaria in humans, *P. falciparum* is the most geographically widespread species and is responsible for the majority of the infections, severe cases, and deaths of malaria [31,32]. *P. falciparum* is the species responsible for the most severe manifestations of malaria, commonly referred to as severe malaria, and is also the species that has developed resistance to virtually all antimalarials currently in clinical use [33].

### 2.1. Epidemiology Data

As recorded in ancient texts, malaria is a cyclical febrile disease. Today, it is known that it is caused by protozoa of the genus *Plasmodium* and transmitted by mosquitoes of the genus *Anopheles*. This genus comprises more than 200 species, but only *P. falciparum*, *P. vivax*, *P. malariae*, *P. ovale*, and *P. knowlesi* affect humans, with the first being the most relevant to public health due to its potential to cause severe cases of malaria [7]. *P. falciparum* was responsible for 77% of global malaria cases, while *P. vivax* affected 21% and the others accounted for approximately 2%. *P. falciparum* is predominant in cases in sub-Saharan Africa. This fact, combined with socioeconomic and environmental issues, helps explain the higher mortality rate in this region. *P. vivax* is more prevalent outside Africa, especially in Asia, Latin America, and the Pacific, while the incidence of other species is rarer globally. For example, *P. knowlesi* is more common in Southeast Asia [34].

In 2023, the World Health Organization reported a global burden of 263 million people affected by malaria, with an incidence of 60.4 cases per 1000 population and an estimated 597,000 deaths worldwide. These numbers represent an increase of 11 million cases compared to 2022 and an increase in incidence from 58.6 to 60.4 cases per 100,000 population. The African continent remains the most affected region, accounting for 94% of cases and 95% of global deaths. In 2023, five countries accounted for the highest malaria burden: Nigeria (26% of global cases or approximately 68.4 million people affected), the Democratic Republic of the Congo (13%), Uganda (5%), Ethiopia (4%), and Mozambique (4%) [34].

In Brazil, the Amazon region accounts for a significant number of malaria cases, with the states of Amazonas (41.9%), Roraima (26.6%), and Pará (17.0%) having the highest concentration of people affected by the disease. Thus, these data represent more than 80% of Brazilian cases, while the rest are directly related to the Amazon region [35].

### 2.2. Life Cycle and the Importance of Subtilisin

**Figure 1:**
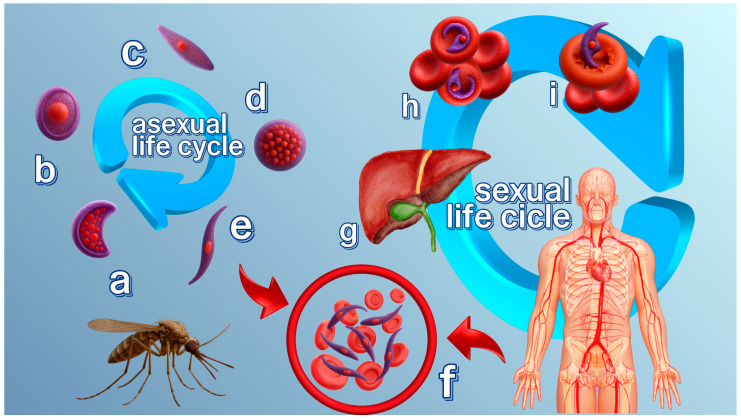
Schematic representation of the life cycles of *Plasmodium* spp. Legend: (**a**) Mature gametocytes; (**b**) zygote; (**c**) ookinete; (**d**) ookinete; (**e**) sporozoites; (**f**) sporozoites in the blood; (**g**) invasion of hepatocytes; (**h**) merozoites invading RBCs; (**i**) replication in trophozoites and schizonts.

The life cycle of the malaria parasite is a complex process involving an Anopheles mosquito and a vertebrate host (Figure 1) [36]. Mature gametocytes (Figure 1a) are taken up by female *Anopheles* spp. mosquitoes during a blood meal, leading to the sporogonic phase of the parasite’s life cycle. In the mosquito midgut, female and male gametes fuse to form the zygote (Figure 1b), which then develops into the ookinete (2n) (Figure 1c). Ookinetes traverse the midgut epithelium of the mosquito and develop into oocysts (Figure 1d) on the basal side of the midgut. Upon maturation, oocysts rupture, releasing thousands of sporozoites (Figure 1e) into the mosquito’s hemolymph. These sporozoites subsequently migrate to the salivary glands, from where they are transmitted to a new human host during the mosquito’s subsequent blood meal (Figure 1f) [37].

The first stage of the infection involves the entrance of sporozoites into the skin and bloodstream of the human host, followed by their invasion of hepatocytes (Figure 1g). This leads to asexual replication and, eventually, the release of thousands of merozoites that invade red blood cells (RBCs) (Figure 1h) [38]. Sequential transformations from rings characterize the asexual replication cycle of *P. falciparum* in erythrocytes to trophozoites and schizonts (Figure 1i) [39]. Merozoites, which are formed and mature within schizonts, egress from the erythrocytes and reinvade new RBCs and continue the asexual replication cycle [40].

During invasion, the initial attachment to the RBC surface is mediated by merozoite surface proteins (MSPs), such as MSP1 [41]. MSP1 is the most abundant merozoite surface protein anchored on the merozoite surface [42]. It essentially mediates erythrocyte invasion via interactions with host glycophorin A [43] and heparin-like molecules [44]. The posttranslational modification and processing of MSP1 by the parasite protease subtilisin-1 (SUB1), which is released from dense granules (specialized secretory organelles) [45], are necessary steps in merozoite maturation [46]. Initially, expressed as a high-molecular-weight protein, MSP1 undergoes primary proteolytic processing [18], resulting in a protein complex that mediates the initial attachment of merozoites to RBCs, facilitating successful invasion [47]. Following egress from the host RBC, MSP1 is further cleaved by *P. falciparum* SUB2, resulting in the shedding of the majority of the MSP1 complex [48]. The precise timing and spatial regulation of these processing events are governed by the discharge of SUB1 [49], which is activated by plasmepsin X through the cleavage of SUB1 inhibitory segments [50]. Genetic depletion of the membrane-bound protease SUB2 disrupts invasion, leading to developmental arrest [51]. Other proteins and protein complexes are also involved in the egress and reinvasion of RBCs [52].

Following initial attachment, the merozoite reorients itself so that its apical end faces the RBC membrane, and a tight junction is formed [53]. With the tight junction established, the merozoite actively invades the RBC, leading to the formation of a parasitophorous vacuole (PV), followed by extensive modification of the host cell to support intracellular development of the parasite [54].

During blood-stage development, a subset of parasites differentiates into gametocytes, the sexual forms responsible for transmission to the mosquito vector [55]. Upon maturation, gametocytes are ingested by female mosquitoes during a blood meal, initiating the mosquito stage of the parasite’s life cycle, completing the transmission cycle.

### 2.3. Clinical Manifestations

The incubation period for malaria varies according to the species: 8 to 12 days for infections involving *P. falciparum*, 13 to 17 days for *P. vivax*, and 18 to 30 days for *P. malariae*. However, not all parasites develop quickly; some remain in the liver in a latent form, such as hypnozoites, which are directly related to relapses of the disease within the first six months after treatment [35].

Acute malaria attacks typically begin with episodes of chills, fever, and sweating that last for 6 to 12 h, accompanied by a body temperature of 40 °C or higher. This is attributed to the rupture of erythrocytes, which release merozoites that invade new red blood cells, produce hemozoin, and release parasitic antigens. This immune response activates macrophages, dendritic cells, and T lymphocytes, producing inflammatory cytokines such as tumor necrosis factor-alpha and interleukins 1 and 6. In the hypothalamus, these cytokines raise the body’s thermal set point, causing vasoconstriction, muscle tremors, and a sensation of cold [56,57].

When the body reaches the new set point and the temperature rises rapidly, symptoms such as headache, nausea, tachycardia, and prostration appear. Cytokine levels then fall, the temperature point adjusts, and the body begins to dissipate heat through vasodilation and sweating, causing the temperature to drop. It is worth noting that the classic pattern of fever every two days, reported since ancient times, is not always observed in all patients. Therefore, one should not wait for this cycle to begin before initiating diagnosis or treatment [58]. Risk groups, such as children, pregnant women, and people with a first-time infection, tend to develop more severe cases of malaria and, for this reason, require medical and pharmaceutical monitoring, especially in cases of *P. falciparum* [59,60].

### 2.4. Antimalarial Drugs

As malaria has been a problem since ancient times, it is to be expected that ancient populations would have been aware of therapies based on medicinal plants. For example, indigenous peoples were already familiar with *Cinchona* spp., a plant belonging to the *Rubiaceae* family, characterized by large shrubs or small trees, varying in height from 5 to 20 m, and used it in the event of tertian fever. The bark of the stem is rich in alkaloids, including quinolines such as quinine and cinchonine, which exhibit antimalarial, anticancer, antioxidant, antidiabetic, antifungal, antimicrobial, and other beneficial activities [61].

**Figure 2:**
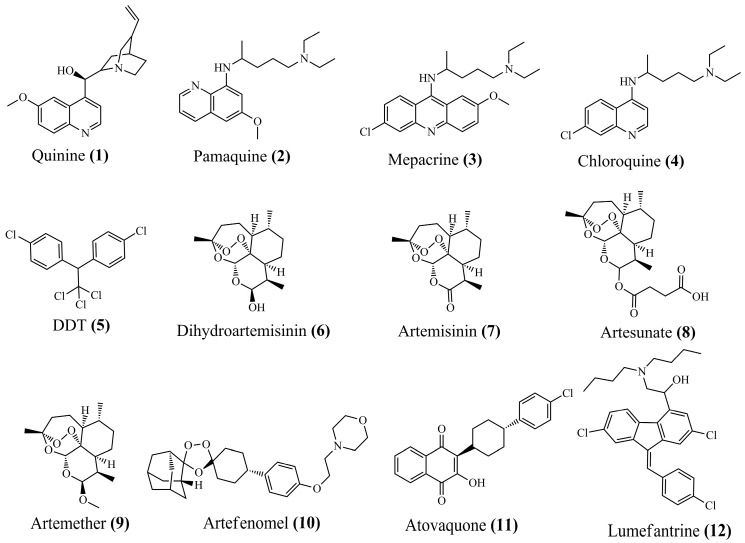
Chemical structure of the main antimalarial drugs.

It was not until 1820 that quinine **(1)** (Figure 2) was isolated and identified as one of the main alkaloids responsible for antimalarial activity. Its structure is composed of a quinoline ring linked to an ether group in position 6 and a small carbon chain linked to a secondary hydroxyl group and a non-aromatic ring with a heteroatom, the quinuclidine ring. Its mechanism is related to interference in the heme metabolism of *Plasmodium* spp. during the intraerythrocytic phase, preventing the detoxification of free heme, leading to the accumulation of toxic substances [61,62]

It was only during the Second World War that synthetic antimalarials emerged: pamaquine **(2)** (1924); mepacrine **(3)** (1930); chloroquine **(4)** (1934); dichlorodiphenyltrichloroethane—DDT **(5)** (1942); and dihydroartemisinin **(6)** (1973) (Figure 2). The first, pamaquine **(2)**, is a derivative of 8-aminoquinoline developed to combat malaria in soldiers, especially in tropical regions. This synthetic derivative exhibits activity against hepatic forms and gametocytes of *Plasmodium* spp., particularly *P. vivax* and *P. ovale*, as well as other members of this class. To improve chemical stability, a salt with a better pharmacokinetic profile was developed, which is effective against hypnozoites and thereby prevents relapses or recurrences. Unlike 8-aminoquinoline derivatives, 4-aminoquinoline derivatives, such as chloroquine **(4)**, are effective against the erythrocytic forms of malaria. However, they do not affect hepatic forms, meaning that these effects do not impede recovery, especially in infections involving *P. vivax* and *P. ovale* [63].

Mepacrine **(3)**, another derivative that shares a quinoline structural relationship with the 4-aminoquinoline derivatives, also known as quinacrine, was developed six years after pamaquine **(2)**, which was derived from methylene blue, a compound that had shown antimalarial activity as early as 1891. This acridine derivative has a general structure similar to other quinolines and is used as a synthetic alternative to quinine **(1)**. Its mechanism of action has not yet been fully elucidated, but it is believed to inhibit the formation of hemozoin, leading to the accumulation of toxic heme [64].

Four years later, chloroquine was developed to treat all forms of malaria, with few side effects. It is structurally based on quinoline, substituted at position 3 and with a chlorine at position 7. As an antimalarial drug, it acts by raising the pH of *Plasmodium* lysosomes, inhibiting the fusion of autophagosomes with lysosomes and also the degradation of lysosomal proteins, thus interfering with the degradation of hemoglobin. Specifically, it inhibits the enzyme heme polymerase, preventing the conversion of toxic heme into non-toxic hemozoin [65].

It was not until 1942, 10 years after the emergence of chloroquine **(4)**, that DDT **(5)** was developed, the first synthetic insecticide to combat malaria vectors, specifically *Anopheles*. This insecticide acts directly on the nervous system of insects, interfering with the sodium channels of neurons and causing hyperexcitation, convulsions, and death in the mosquito. However, it is highly persistent in the environment and can accumulate in the tissues of animals and humans. For this reason, its sale in the agricultural market is prohibited, although its use in public health remains restricted [66,67,68].

For over 30 years, researchers have been searching for new antimalarials due to the resistance mechanisms that *Plasmodium* has developed against chloroquine **(4)**. With these efforts, *Artemisia annua* was identified in Chinese medicine as a plant whose extract contains artemisinin **(7)** (Figure 2), a sesquiterpene lactone endoperoxide. Its oxygen bridge between the cycles is directly related to the capture of Fe^2+^, forming free radicals that are toxic to the parasite [69,70]. A series of derivatives emerged from artemisinin **(7)**: dihydroartemisinin **(6)**, artesunate **(8)**, artemether **(9)**, and artefenomel **(10)** (Figure 2). All of these derivatives have a mechanism similar to artemisinin **(7)**, differing in indications, stability, forms of administration, safety, and other pharmacokinetic aspects. [69,70,71,72,73,74,75].

In response to the growing resistance to chloroquine **(4)**, especially in the treatment of *Plasmodium falciparum* infections, drugs such as atovaquone **(11)** and lumefantrine **(12)** (Figure 2) have been developed and introduced in combination with artemisinin derivatives, such as artesunate **(8)**, artemether **(9)**, and artefenomel **(10)**. These combinations aim to explore distinct mechanisms of action, allowing them to act at multiple stages of the parasite’s evolutionary cycle and, at the same time, reduce the risk of developing resistance. For example, artemisinin **(7)** and its derivatives promote a rapid reduction in parasitemia in the first hours of treatment but have a short half-life, which limits their prolonged action. In this context, the combination with lumefantrine **(12)**, which has a long half-life, enables therapeutic concentrations to be maintained for a more extended period, ensuring sustained efficacy. Furthermore, while the use of monotherapy favors the selection of resistant strains, the use of therapeutic combinations significantly reduces the likelihood of simultaneous emergence of mutations that would confer resistance to both drugs [76,77,78,79].

Although the development of therapies for chronic non-communicable diseases, such as diabetes mellitus and systemic arterial hypertension, has progressed more rapidly, it is clear that malaria therapy has evolved over the years to meet the needs related to the particularities of the species, the stage of infection, and population groups, while considering both safety and effectiveness. This is due to the fact that in many contexts, malaria mainly affects low-income countries, receives little investment in research and development, and relies on old control methods [80,81,82].

### 2.5. Druggable Targets Against Malaria

To discover new antimalarial drugs, several targets and enzymes essential for the metabolism and survival of *Plasmodium falciparum* have been explored, mainly to overcome resistance. Among them are dihydrofolate reductase [83,84], dihydropteroate reductase [84], plasmepsins, and facipains [85,86].

Dihydropteroate reductase (DHPS) is an essential enzyme for folate biosynthesis in *Plasmodium* spp., acting in a step prior to DHFR, which catalyzes the conversion of para-aminobenzoic acid (PABA) to dihydropteroate, a crucial precursor for the production of tetrahydrofolate, a cofactor essential for the synthesis of nucleic acids and amino acids. Unlike humans, *Plasmodium* relies entirely on these endogenous pathways for folate synthesis; however, it is worth noting that mutations can generate resistance to inhibitors of the DHPS and DHFR pathways [84].

Dihydrofolate reductase (DHFR) is another essential enzyme for *Plasmodium* spp., as it participates in the synthesis of folate, a fundamental metabolite required for the production of purines and thymidylate, which are necessary for DNA replication and cell division in the parasite. During the life cycle, especially in the phases of intense replication, such as the hepatic and erythrocyte phases, the demand for DNA synthesis is high, making it a critical target for the survival of the parasite [83].

Plasmepsins also stand out for their involvement in the survival of the protozoan during the erythrocyte phase and in the symptoms. They are a group of aspartyl protease enzymes specific to *Plasmodium* spp., which cleave hemoglobin, allowing other proteases to continue the degradation process. Furthermore, due to the absence of homologues in humans, plasmepsins become attractive targets in the development of new antimalarials [86].

Regarding the steps of hemoglobin degradation by the protozoan, falcipains, papain-type cysteine protease enzymes, also stand out as druggable antimalarial targets. Acting together with plasmepsins, they release peptides and amino acids from hemoglobin, which the parasite uses for protein synthesis and the maintenance of its metabolism. Their isoforms 2 and 3 are the most extensively studied and considered vital for the parasite’s survival in erythrocytes. In addition, it is worth noting that humans have similar proteases to falcipains, and the target specificity is a challenge to overcome in the exploration of this drug target [85,86].

Falcilysins, like plasmepsins, are metalloproteins primarily found in the digestive vacuole of *Plasmodium*, where they participate in the degradation of hemoglobin, a crucial process for the parasite’s nutrition. However, they have also been identified in mitochondria and the apicoplast, indicating a multifunctional role. Inhibition of these enzymes represents a promising strategy, as it directly compromises the erythrocyte forms of the parasite, especially the trophozoites, the stage in which intense metabolic activity occurs [79].

Another protease expressed by *Plasmodium falciparum*, subtilisin, a serine protease, is a target for the development of synthetic drugs against malaria. This enzyme acts in the final stage of the erythrocyte cycle, responsible for cleaving proteins involved in the rupture of the parasitophorous vacuole and red blood cell membranes, thereby enabling the release of merozoites and facilitating progression to other red blood cells. The stages directly affected by this enzyme are mature schizonts and merozoites in release; that is, inhibitors for this serine protease compromise the rupture of the red blood cell and block the spread of the parasite [18]. Thus, the next topic will explore the importance, structure, and function of subtilisin, with a focus on designing new inhibitors for drug development.

## 3. Subtilisin: A Promising Drug Target

Subtilisin (SUB) is a serine protease that belongs to the subtilase family, a primordial group of proteolytic enzymes conserved across all domains of life [18,87]. Through evolution, this protease family diversified, and in eukaryotes, it gave rise to subtilisin-like proteases, which retain the characteristic serine protease catalytic mechanism, with the hydroxyl group of the catalytic serine acting as a nucleophile [18,87].

In *P. falciparum*, three subtilisin-like serine proteases (SUB1, SUB2, and SUB3) were identified, each performing distinct and essential roles in the parasite’s life cycle, including merozoite egress and red blood cell invasion. Although *P. falciparum* and other eukaryotes possess these SUB enzymes, humans instead have a related but distinct family of subtilisin-like proteases known as proprotein convertases (PCs). These PCs belong to the subtilase subfamily and are involved in the proteolytic activation of diverse precursor proteins. However, they are structurally and functionally distinct from *Plasmodium* SUB enzymes [87,88,89]. Notably, no close human homolog of SUB1 has been identified. The human protease furin shares 21.7% sequence identity with SUB1 and differs in substrate specificity, favoring dibasic motifs at the **P2**–**P1** positions [18]. Another human subtilisin-like enzyme, proprotein convertase subtilisin/kexin type 2 (PCSK2), shares 34.1% identity with SUB1. PCSK2 is a subtilisin-like protease involved in processing protein and peptide precursors within the regulated and constitutive secretory pathways [90]. In addition, a brief search using the BLAST-2.17.0 tool by NCBI (https://blast.ncbi.nlm.nih.gov/, accessed on 23 July 2025) revealed no similarity between SUB2 and humans or between SUB3 and humans.

Given the absence of close human homologs, inhibitors of *Plasmodium falciparum* subtilisin-like proteases represent promising antimalarial drug candidates, with a low likelihood of off-target effects [18]. Such compounds could provide a novel and broadly effective addition to antimalarial therapies. Finally, due to the importance of these enzymes, in the following section, we will highlight the structure and function of these targets to gain a deeper understanding of their potential for therapeutic intervention.

### 3.1. Structure and Functions

Subtilases comprise a functionally diverse group of serine proteases that primarily operate in the secretory pathway or extracellular sites [87]. Subtilases are distinguished from other serine proteases by the order in which their catalytic triad residues are arranged: aspartate, histidine, and serine (Asp-His-Ser) [87]. These catalytic residues are flanked by conserved sequence motifs that are readily identifiable, facilitating their classification within the subtilase family [87]. In *Plasmodium falciparum*, all three subtilisin-like proteases are expressed during the asexual blood stages, and two of them (SUB1 and SUB2) have been associated with specialized secretory organelles within the invasive merozoite, underscoring their roles in parasite egress and invasion. Further sections will explore in detail the structural features and functional diversity of these proteases [87,91].

#### 3.1.1. SUB1

*Plasmodium falciparum* subtilisin-like protease 1 (SUB1) is a key regulator of parasite egress during the asexual blood stage of malaria. First identified through PCR-based screening for subtilisin-like serine proteases in the parasite genome [92], SUB1 drives the sequential rupture of the parasitophorous vacuole membrane (PVM) and the host erythrocyte plasma membrane, enabling merozoite release and the propagation of infection [18,93].

SUB1 is synthesized on ribosomes associated with the endoplasmic reticulum (ER) as a full-length 82-kDa zymogen (p82) directed into the secretory pathway by an N-terminal signal peptide [18]. The zymogen comprises an N-terminal prodomain (PD), which acts as an intrinsic inhibitor and folding chaperone, and a C-terminal catalytic domain [94]. The prodomain adopts a substrate-like conformation, with its C-terminal segment spanning the enzyme’s substrate-binding groove (P4–P1), forming an antiparallel β-sheet that sterically blocks the active site [94]. As SUB1 transits from the ER to the Golgi, it undergoes two sequential proteolytic maturation steps. The first autocatalytic cleavage occurs at specific internal aspartate residues, producing a 54-kDa intermediate (p54) that remains transiently bound to a 31-kDa prodomain fragment (p31). This initial processing relieves autoinhibition, allowing SUB1 to adopt a catalytically competent conformation necessary for trafficking [18]. A second autocatalytic cleavage generates the mature 47-kDa form (p47), which is stored in specialized secretory vesicles called exonemes [93]. While autocatalysis mediates these cleavages, the aspartic protease plasmepsin X (PMX) has been implicated in regulating SUB1 activation, though the mechanism remains unclear [50].

**Figure 3:**
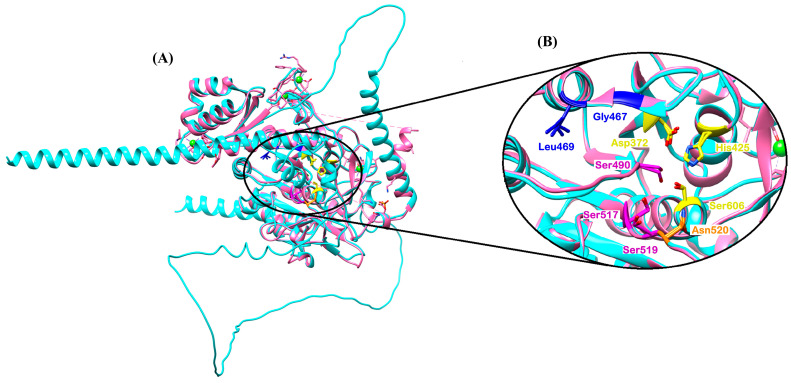
Three-dimensional structure of the SUB1: (**A**) Structural alignment of the full-length SUB1 protein (PDB ID: 8POL) (in magenta) with its predicted AlphaFold model based on the SUB1 FASTA sequence (in cyan), visualized in UCSF Chimera. (**B**) The catalytic groove is zoomed in to show the catalytic groove, which results in the formation of a two-stranded antiparallel *β*-sheet. Key functional residues mapped onto the SUB1 structure, Leu^469^ and Gly^467^ (blue), defining part of the hydrophobic **S4** substrate-binding pocket; the catalytic triad (Asp^372^, His^428^, Ser^606^) essential for proteolytic activity (yellow); Ser^490^, Ser^517^, and Ser^519^ (magenta), forming part of the polar **S1** pocket; and Asn^520^ (orange), which contributes to the oxyanion hole stabilizing the catalytic transition state.

Structurally, SUB1 belongs to the S8 family of serine proteases, adopting a conserved α/β fold and housing a catalytic triad (Asp^372^–His^428^–Ser^606^) essential for peptide bond hydrolysis. In this way, Asp^372^ polarizes His^428^ to activate the nucleophile Ser^606^ for catalysis [18]. A complementary oxyanion hole, formed by Asn^520^, stabilizes the negatively charged tetrahedral intermediate during the transition state, ensuring efficient catalysis [18]. The crystal structure (PDB ID: 8POL) (Table 1) has revealed uniquely configured substrate-binding pockets. The **S1** pocket is highly polar, engaging acidic **P1** residues via Ser^490^, Ser^517^, and Ser^519^ (Figure 3). The hydrophobic **S4** pocket, which features residues Gly^46^ and Leu^469^ (Figure 3), favors aliphatic side chains. These pockets collectively determine *Pf*SUB1 substrate specificity. On the prime side of the scissile bond, a large basic **S′** pocket stabilizes interactions at **P1′**–**P3′**, further enhancing substrate recognition [18].

Environmental factors also modulate SUB1 activity. At acidic pH, it undergoes calcium-dependent activation and homodimerization, while at neutral pH (as in the PV lumen), it predominantly exists as an active monomer [95]. A redox-sensitive disulfide bond (Cys^521^–Cys^534^), resolved in structural studies, likely provides additional regulation by acting as a conformational switch near the active site [96]. In the erythrocytic cycle, SUB1 plays a central role in initiating egress. Schizogony within infected red blood cells produces merozoites enclosed in the PV. Approximately 10 min before merozoite release, intracellular calcium oscillations activate cyclin-dependent protein kinase 5 (CDPK5), followed by cGMP-dependent protein kinase G (PKG) activation. These kinases coordinate the release of SUB1rom exonemes into the PV lumen [50]. Once secreted, SUB1 cleaves and activates several substrates, including members of the serine-rich antigen (SERA) family, leading to PVM disassembly and rupture [18]. This process releases merozoites into the erythrocyte cytosol, from which they are subsequently released upon rupture of the host-cell plasma membrane.

SUB1 is thus indispensable for merozoite egress and represents a promising target for novel antimalarial interventions. It is precisely orchestrated maturation, activation, and substrate processing that underscore its critical role in the asexual blood-stage lifecycle.

#### 3.1.2. SUB2

**Figure 4:**
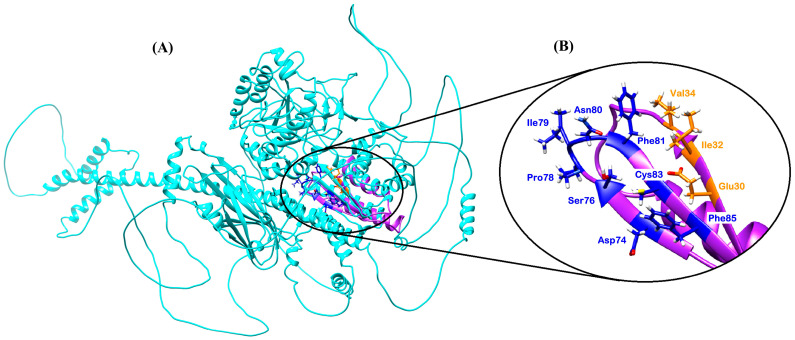
Three-dimensional structure of the SUB2 in (**A**) the structural alignment of the catalytic domain of SUB2 protein (PDB ID: 2LU1) (purple) with its full-length predicted AlphaFold model (cyan) based on the SUB2 protein sequence available in NCBI (ID: XP_001348051.1), which shared 99% identity with the PDB ID 2LU1 sequence. (**B**) Key predicted structural features of SUB2 are highlighted: Val^34^, Ile^32^, and Glu^30^ (orange), which contribute to the β1 strand; Asp^74^ and Ser^76^ (blue) form part of the β2 strand; Pro^78^, Ile^79^, and Asn^80^ (blue) constitute a connecting loop; and Cys^83^ and Phe^85^ form the β3 strand. Together, the β2–β3 strands and intervening loop adopt a hairpin-like topology, which may contribute to substrate recognition and catalytic stability.

*Plasmodium falciparum* subtilisin-like protease 2 (SUB2) (Figure 4) is an essential membrane-bound serine protease that plays an essential role in both invasion and egress during the asexual blood-stage cycle. Unlike SUB1, which operates in the parasitophorous vacuole, SUB2 is trafficked to the parasite plasma membrane and secretory organelles, where it executes key proteolytic steps necessary for merozoite maturation and host-cell invasion [97].

SUB2 is synthesized as a ~170 kDa zymogen comprising a large N-terminal prodomain and a C-terminal catalytic domain. The prodomain acts as an intramolecular chaperone, preventing premature activation during trafficking through the secretory pathway. Cleavage and removal of the prodomain yield the mature active protease (p150), which localizes predominantly to micronemes and the merozoite surface [97]. Initially, SUB2 accumulates in a set of apical secretory organelles called micronemes, which are the destination of other invasion factors such as erythrocyte-binding antigens and apical membrane antigen 1 (AMA1) [97,98]. Following merozoite egress, SUB2 is discharged from these organelles and traffics across the surface of the free merozoite in an actin-dependent way, enabling redistribution to the parasite’s posterior pole [99].

Although no crystal structure is available for full-length SUB2, structural insights into its catalytic domain have been obtained through an NMR model (PDB ID: 2LU1) (Table 1), which represents a truncated fragment of approximately 150 amino acids from the C-terminal region of the protein [100]. The catalytic activity of SUB2 is mediated by the conserved triad of Asp, His, and Ser residues, which align spatially within the truncated domain to facilitate peptide bond hydrolysis [100].

The SUB2 function is defined by its ability to shed adhesins from the parasite surface, a process critical for merozoite motility and erythrocyte invasion. Merozoite surface protein 1 (MSP1) is cleaved near its C-terminus to remove large surface complexes prior to invasion. Apical membrane antigen 1 (AMA1) is processed at its juxtamembrane region to facilitate tight junction formation during entry. Additional adhesins, including members of the erythrocyte-binding antigen (EBA) family, are released from the merozoite surface in a SUB2-dependent manner, coordinating the rapid transition from egress to invasion [97].

These proteolytic events, collectively referred to as merozoite shedding, are crucial for the efficient invasion and immune evasion of the parasite. Genetic knockdown or pharmacological inhibition of SUB2 results in profound defects in both merozoite egress and host-cell invasion, underscoring its indispensability for blood-stage replication [97]. In addition, similar to SUB1, SUB2 exhibits calcium-dependent regulation, with activation coinciding with microneme secretion that is critical for the processing of egress and invasion of specific proteins [101]. However, its membrane-anchored architecture and distinct substrate repertoire reflect its specialized role at the host–parasite interface [97]. Together, these properties position SUB2 as a promising target for transmission-blocking antimalarial strategies.

#### 3.1.3. SUB3

Among the subtilisin-like proteases of *Plasmodium falciparum*, SUB3 remains the least characterized, yet it presents intriguing potential as a drug target. Unlike SUB1 and SUB2, which are essential for merozoite egress and invasion, SUB3 appears to play a more nuanced role in the parasite’s life cycle. Gene disruption studies have shown that SUB3 is dispensable for asexual blood-stage development in vitro, suggesting it may not be critical for erythrocytic replication. However, transcriptomic analyses reveal that SUB3 is expressed during both blood stages and sexual differentiation, with particularly high expression in gametocytes and sporozoites [87,102,103]. This pattern implies a possible role in transmission or liver-stage development, which are less explored but equally vital for malaria eradication strategies [102,104]. Structurally, SUB3 shares greater homology with SUB1 than with SUB2, particularly in its catalytic domain, and lacks a transmembrane domain, suggesting it may function in a soluble or secreted form [87,103]. Although no crystal structure is currently available, sequence analysis indicates the presence of a conserved catalytic triad (Asp-His-Ser), characteristic of subtilisin-like serine proteases. Interestingly, SUB3 lacks a classical signal peptide, which raises questions about its trafficking and activation mechanisms. If proteolytically active, the presence of a downstream phenylalanine residue near the scissile bond may influence substrate specificity, potentially distinguishing it from SUB1 and SUB2 [104]. Given its expression in transmission stages and structural divergence from human homologs, SUB3 represents a promising, albeit underexplored, target for transmission-blocking interventions. Further biochemical and structural studies are essential to elucidate its function and validate its druggability.

Gene disruption studies in *P. falciparum* indicate that SUB3 is dispensable for in vitro asexual blood-stage growth [18]. No crystal structure is available; however, microarray analyses reveal transcription during blood stages and significant expression in gametocytes and sporozoites [87]. Structurally, SUB3 resembles SUB1 more than SUB2, lacking a membrane-spanning domain and annotated in PlasmoDB as without a classical secretory signal, although most subtilases are secreted [87]. Proteolytic activity has not been experimentally confirmed, but if active, the downstream Phe residue may influence substrate recognition [87].

**Table 1:** Summary of primary sequence information on the *P. falciparum* subtilases.

Subtype	PDB ID ^a^	Gene ID ^b^	Number of Amino Acid Residues in Modeled Protein (and Predicted M_r_)
*Pf*SUB1	8POL	-	688 (78 KDa)
*Pf*SUB2	2LU1 (catalytic structure)	-	150 (17KDa) (catalytic structure)
*Pf*SUB3	-	PFE0355c	769 (88 KDa)

^a^ refers to Protein Data Bank [105]. ^b^ refers to annotated gene ID as used in PlasmoDB v4.1 (http://plasmodb.org/, accessed on 23 July 2025).

### 3.2. Catalysis Mechanism

**Figure 5:**
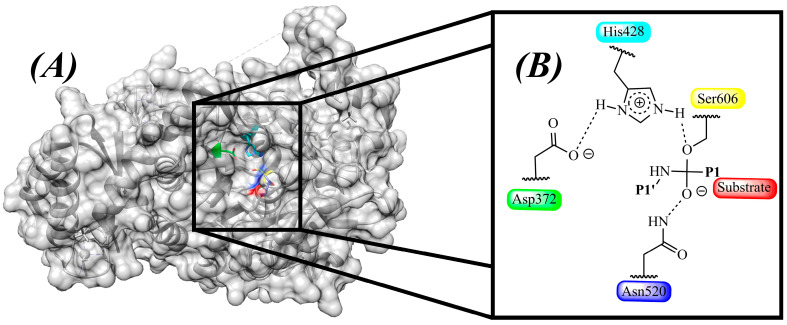
Hydrophobic surface of SUB1 (**A**), highlighting the catalytic residues (**B**), composed of Asp^372^, His^428^, Ser^606^, and the critical residue for binding substrate Asn^520^.

The conserved serine in the catalytic triad mediates catalysis in SUB1: Asp^372^ (acid), His^428^ (base), and Ser^606^ (nucleophile) (Figure 5). An auxiliary residue, Ser^50^, may contribute a weak hydrogen bond to the catalytic base, potentially stabilizing His^428^ during the catalytic process [106]. Additionally, Asn^520^ forms part of the oxyanion hole, which stabilizes the tetrahedral intermediate of the substrate during peptide bond hydrolysis [106]. This mechanism provides critical information that can be used in drug design. For example, researchers worldwide can focus on this mechanism to design new drugs that interfere with the catalysis and inhibit it, thereby stopping the growth of the parasite. Furthermore, this information can be used in CADD works to design inhibitors with high affinity and greater selectivity.

On the other hand, SUB2, while sharing the same catalytic triad architecture, exhibits distinct substrate preferences and localization. It is membrane-anchored and primarily involved in shedding surface adhesins like MSP1 and AMA1 during erythrocyte invasion. Although structural data are limited to a truncated NMR model, the catalytic domain of SUB2 retains the Asp-His-Ser triad, and its activity is tightly regulated by microneme secretion and actin-dependent trafficking [51,100]. Unlike SUB1, SUB2 operates at the host–parasite interface, and its substrate-binding groove is adapted to accommodate membrane-associated proteins, suggesting a broader and more flexible binding pocket.

SUB3, though less characterized, is predicted to possess the canonical subtilisin catalytic triad based on sequence homology. However, the absence of structural data and experimental confirmation of proteolytic activity leaves its catalytic mechanism speculative. If active, differences in substrate-binding residues, such as the presence of a downstream phenylalanine, may confer unique specificity, potentially targeting substrates involved in gametocyte maturation or sporozoite development [103,107]. Finally, the catalytic mechanism of *Pf*SUB3, while not experimentally resolved at the structural level, appears to follow a classical subtilisin-like serine protease paradigm, as inferred from homology modelling and sequence-based predictions. Its active site includes the Asp–His–Ser triad, a hallmark of subtilisin family enzymes. Computational models assign Ser^701^ as the likely nucleophilic residue poised to attack the substrate’s carbonyl carbon. A nearby histidine, based on sequence alignment, is predicted to act as a general base, accepting a proton from the Ser^701^ hydroxyl group. The final residue of the triad, aspartate, is thought to stabilize the positively charged histidine during catalysis. Although the exact residue numbering may differ slightly from other subtilases, the overall spatial configuration is consistent with the canonical arrangement [23,50,87,94,104,106,108,109,110].

Mechanistically, the model posits that Ser^701^ initiates nucleophilic attack on the substrate’s peptide bond, forming a tetrahedral intermediate. This intermediate is presumed to be stabilized by an oxyanion hole and then collapses to release the amino-terminal peptide and form the acyl–enzyme intermediate. Finally, hydrolysis by a water molecule would regenerate the free enzyme, releasing the carboxyl-terminal fragment.

Importantly, these mechanistic propositions rest solely on in silico analyses, including modelling and sequence agreement, without corresponding structural or mutational validation. As such, the precise roles of the proposed triad residues remain hypothetical pending experimental confirmation through methods such as site-directed mutagenesis or crystallography. So far, the evidence suggests all three proteases use the canonical serine protease mechanism: Ser attacks the peptide carbonyl, forming a tetrahedral intermediate stabilized by the oxyanion hole, followed by acyl-enzyme formation and hydrolysis.

Comparative analysis of the substrate-binding pockets across the three enzymes (Table 2) reveals that SUB1 favors acidic residues at the P1 position, stabilized by polar residues like Ser^490^, Ser^517^, and Ser^519^, while SUB2 appears to accommodate more hydrophobic or membrane-associated substrates. The substrate preferences of SUB3 remain unknown, but its divergence suggests a distinct functional niche. These mechanistic insights are critical for rational drug design, as they enable the development of selective inhibitors that exploit the unique structural features of each enzyme.

## 4. Latest Developments in Medicinal Chemistry to Discover Subtilisin Inhibitors

The development of subtilisin inhibitor compounds with antimalarial activity has, to date, involved a combination of various approaches, ranging from screening small-molecule libraries to the use of advanced rational drug design. Among these, notable efforts include the development of modified peptides and peptidomimetic drugs based on the structure and mechanism of native SUB1 enzyme substrates, incorporating essential functional groups represented by electrophilic ketones [111]. Hence, the following section examines the progress in developing potential antimalarial drugs that target this crucial serine protease.

### 4.1. Peptide and Peptidomimetic Structures

#### 4.1.1. Cystine Knot Protein Scaffolds

Based on the structure of the toxins of *Psalmopoeus cambridgei*, the Trinidad chevron tarantula, Choi et al. [112] prepared two modified natural miniproteins: *Psalmopoeus cambridgei*, ***Pc*FK1**, and *Psalmopoeus cambridgei*, ***Pc*FK2**, containing, respectively, 33 and 28 amino acid residues, both with six cysteine residues forming three disulfide bridges. Next, in the in vitro evaluation, the intraerythrocytic development of the *P. falciparum* parasite was inhibited in both, presenting IC_50_ values of 1.59 ± 1.15 µM and 1.15 ± 0.95 µM, respectively. Additionally, these miniproteins did not exhibit any toxic effects on red blood cells (at 10 µM) and HeLa cells (up to 50 µM and 20 µM, respectively). Therefore, the authors concluded that the modified natural miniproteins interact specifically with erythrocytes infected by *P. falciparum* and encouraged further studies with ***Pc*FK1**, which identified structural determinants common to several neurotoxins acting as ion channel effectors, proposing an ion channel as a possible molecular target.

Given these promising results, Bastianelli et al. [113] propose subtilisin as a potential pharmacological target of the cystine knot ***Pc*FK1**. In silico ensemble docking was performed, indicating an optimal distance of approximately 2.72 Å between the carbonyl carbon of the Gln residue in the ligand and the hydroxyl group of the catalytic serine (S268) in the target. Next, molecular dynamics (MD) simulations showed calculated free energy of binding averaged values of −5.01 kcal/mol, with RMSD < 3 Å. In vitro, ***Pc*FK1** presented a K_i_ value of 29.3 µM in the SUB1 enzyme of *P. falciparum*. This result, if converted into free energy binding, equals −6.37 kcal/mol, a value close to that obtained in silico. Additionally, ***Pc*FK1** inhibits SUB1 of *P. vivax* with a K_i_ of 36.3 µM. Thus, the structural features of ***Pc*FK1** represent an interesting protein scaffold for future protein engineering.

Through a computational approach that included homology modeling, molecular docking, and mutation scoring, Bastianelli et al. [114] utilized the small peptide structure composed of 28 amino acids of **EETI-II**, a trypsin inhibitor extracted from *Ecballium elaterium*, as a scaffold for designing *P. vivax* SUB1 inhibitors. From this scaffold, substitutions were made in the residues involved in binding to the protease catalytic groove, using the sequence of the *Plasmodium vivax* SUB1 (*Pv*SUB1) natural hexapeptide substrate, to form the **EETI-II**-sub mutants. When evaluated in silico, the **EETI-II-P4L-P1W** structure, obtained by two substitutions in the scaffold (valine for leucine in position **P4** and aspartate for tryptophan in position **P1**), showed better stereochemical complementarity with the active site of *Pv*SUB1 because it contains hydrophobic and bulky residues at position **P4** and aromatic residues with polar groups in position **P1**. In vitro, this structure exhibited a K_i_ value of 86 µM in *Pv*SUB1, which is nine times more potent than the **EETI-II** scaffold.

#### 4.1.2. Peptidyl α-Ketoamides

The α-ketoamide fragment is a privileged structure in medicinal chemistry, notable for its versatile nature, which allows it to act as both an electrophile and a nucleophile, featuring two nucleophilic sites and two electrophilic centers, and serving as an essential precursor in the synthesis of organic compounds targeting diverse biological activities [115]. Serine proteases, such as subtilisin, are promising targets for the rational design of drugs containing the α-ketoamide moiety, since α-ketoamide peptides are known to act as covalent inhibitors of these enzymes, typically interacting with the catalytic serine hydroxyl of the substrate by a reversible covalent bond [18,116,117].

**Figure 6:**
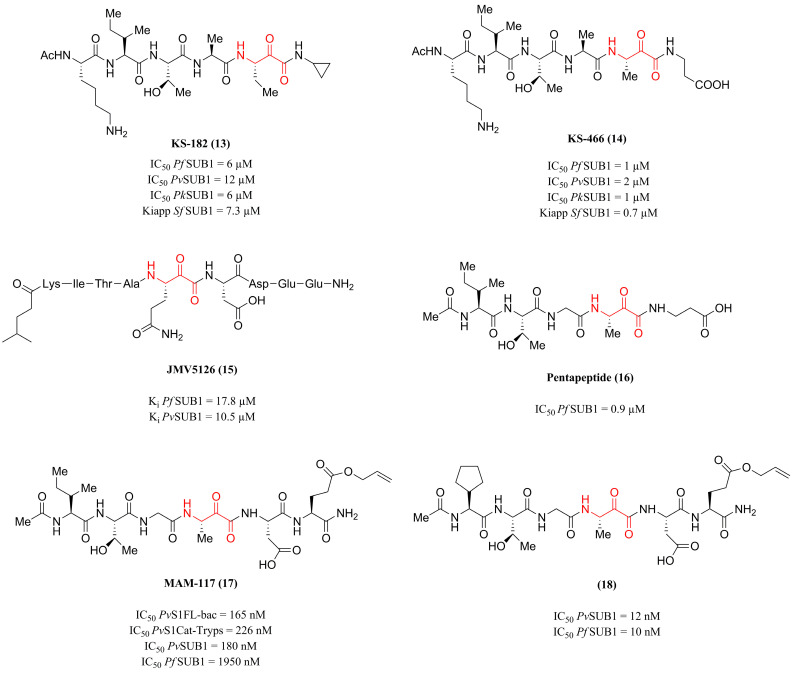
Chemical structures of peptidyl α-ketoamides subtilisin inhibitors.

In this way, Withers-Martinez et al. [18] designed, synthesized, and evaluated the inhibitory activity of two α-ketoamide peptide derivatives against *Plasmodium* sp. subtilisin. Based on the structure of the best-known *P. falciparum* subtilisin substrate, SERA4st1, rational design of the compounds KS-182 **(13)** (absence of prime side residues) and KS-466 **(14)** (Figure 6) (prime-side carboxyl moiety present and designed to mimic the acidic residues) was performed. Biochemical and analytical techniques were used to evaluate the inhibitory activity of these compounds against the subtilisin of *P. falciparum*, *P. vivax*, and *P. knowlesi* (*Pf*SUB1, *Pv*SUB1, and *Pk*SUB1, respectively). Thus, the compounds presented the following approximate IC_50_ values: 6 µM **(13)** and 1 µM **(14)** in *Pf*SUB1, 12 µM **(13)** and 2 µM **(14)** in *Pv*SUB1, and 6 µM **(13)** and 1 µM **(14)** in *Pk*SUB1. When evaluated for their apparent tight binding inhibition constant (Kiapp) in *Pf*SUB1, the compounds show, respectively, values of 7.3 and 0.7 µM. Therefore, the authors highlight the feasibility of developing broad-spectrum drugs capable of inhibiting the SUB1 enzyme of the three main species that cause malaria in humans.

These findings prompted the work developed by Fulle et al. [118] to determine the essential molecular components for the enzymatic inhibition of SUB1 from *Plasmodium* sp. The mapping involved MD simulations in combination with MM-GBSA free energy calculations on a homology model of *Pf*SUB1 in a complex with different substrate peptides. Key interactions were identified at the prime and nonprime side of the scissile bond and comprise peptide residues **P4** to **P2’**, identifying **P4** (⌀Δ*G*_eff_ = −8.2 ± 0.5 kcal/mol) and **P1** (⌀Δ*G*_eff_ = −6.4 ± 1.4 kcal/mol) as hotspot residues, preferentially through van der Waals interactions and the nonpolar part of solvation energy. Therefore, the results enhanced the rational design of peptidomimetic drugs, especially α-ketoamide derivatives, by facilitating effective molecular profiling to complement the complex formation between the molecule and the active site.

Following the same strategy, Giganti et al. [119] synthesized the SERA4-derived peptide JMV5126 **(15)** (Figure 6), featuring an α-ketoamide warhead, aiming at the inhibition of SUB1 of the species *P. vivax* and *P. falciparum*, based on the sequence similarity between the enzymes, especially in the residues that compose the catalytic site. Compound **(15)** was obtained by replacing the **P1**–**P10** scissile peptide bond of SERA4 with an α-ketoamide center. It showed the capacity to inhibit both enzymes (*Pf*SUB1 and *Pv*SUB1) with equivalent K_i_ of 17.8 ± 2.9 and 10.5 ± 1.6 µM, respectively. These findings demonstrate the conservation of the SUB1 active site between species and reinforce the feasibility of developing a pan-species antimalarial drug.

In the work developed by Kher et al. [120], nineteen α-ketoamide derivatives were proposed based on the KITAQ↓DDEES sequence derived from SERA proteins. These compounds were evaluated against the *P. falciparum* SUB1 protease, enabling the establishment of a structure–activity relationship (SAR) through the substitution of amino acid residues in different portions of the structure. Pentapeptide **(16)** (Figure 6), developed with substitutions in positions **P2**, **P3** and **P4** with the amino acids glycine, threonine, and isoleucine, respectively, emerged as the most promising compound by exhibiting an IC_50_ of 0.9 µM in *Pf*SUB1, in addition to standing out for being a less complex and less polar structure compared to the initial prototype of the series.

Therefore, using compound **(17)** (Figure 6) as a reference compound for the development of 40 new α-ketoamide derivatives, Legru et al. [111] used molecular modification strategies to determine the essential structural components for SUB1 inhibition. In this way, derivative **(18)** (Figure 6) stood out as a potent inhibitor, presenting IC_50_ values of 12 nM (*Pv*SUB1) and 10 nM (*Pf*SUB1), evidencing a significant improvement in inhibitory activity compared to **(17)** (IC_50_ = 180 nM and 1950 nM, respectively) caused by the substitution of amino acid residues at positions **P4** (isoleucine replaced by cyclopentylglycine) and **P2’** (glutamine replaced by cyclopentylglycine), increasing the overall hydrophobicity of the compound. Additionally, compound **(18)** inhibited merozoite egress by 100% at a concentration of 100 µM, by 60% at a concentration of 25 µM, and presented an IC_50_ value of 23 µM on *P. falciparum* merozoite egress. In addition, when evaluated against human serine proteases (trypsin, chymotrypsin, and elastase), compound **(18)** did not demonstrate significant inhibition. Thus, the findings highlight the potential of compound **(18)** as a prototype for future studies focused on optimizing its permeability and pharmacokinetic profile.

**Figure 7:**
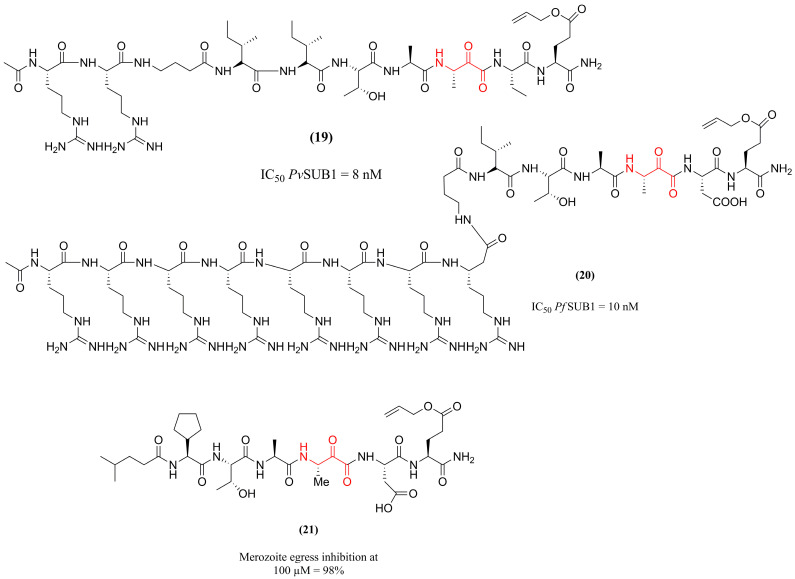
Peptidic α-ketoamides inhibitors of plasmodial SUB1 evaluated by Puszko and coworkers (2025).

Continuing this investigation, Puszko et al. [121] sought to enhance the antiparasitic activity of compounds **(17)** and **(18)** (see Figure 6) by substituting the N-terminal acetyl group with various basic or hydrophobic moieties and by introducing conformational constraints through cyclization. The evaluation of the enzymatic inhibition of 40 monomers and 10 cyclic dimers against the proteases *Pv*SUB1 and *Pf*SUB1 identified two compounds as having greater potency for each isoenzyme: compound **(19)** (IC_50_ = 8 nM for *Pv*SUB1) and **(20)** (IC_50_ = 10 nM for *Pf*SUB1) (Figure 7). The authors inferred that the peptide extension helped to establish additional interactions between the enzyme and these inhibitors. However, the antiparasitic activity profile was enhanced by adding hydrophobic alkyl chains (isocaproyl), such as compound **(21)** (Figure 7), which inhibited parasite growth by 98% at 100 µM. Thus, the study concluded that adding hydrophobic groups or using octaarginine as a cellular carrier are essential strategies to increase cellular penetration. These results provide insight for future studies aimed at improving the pharmacokinetics of the derivatives.

#### 4.1.3. Peptidyl Difluorostatones

Difluorostatones are fluorinated analogs of statins known for their potent protease inhibitory properties. Their inhibitory mechanism is attributed to the presence of a highly electrophilic carbonyl, whose reactivity is enhanced by the electronic effect of adjacent fluorine atoms. This carbonyl is susceptible to nucleophilic attack by the catalytic serine of the target, resulting in the formation of a thermodynamically stable tetrahedral hemiketal intermediate that blocks enzymatic activity.

**Figure 8:**
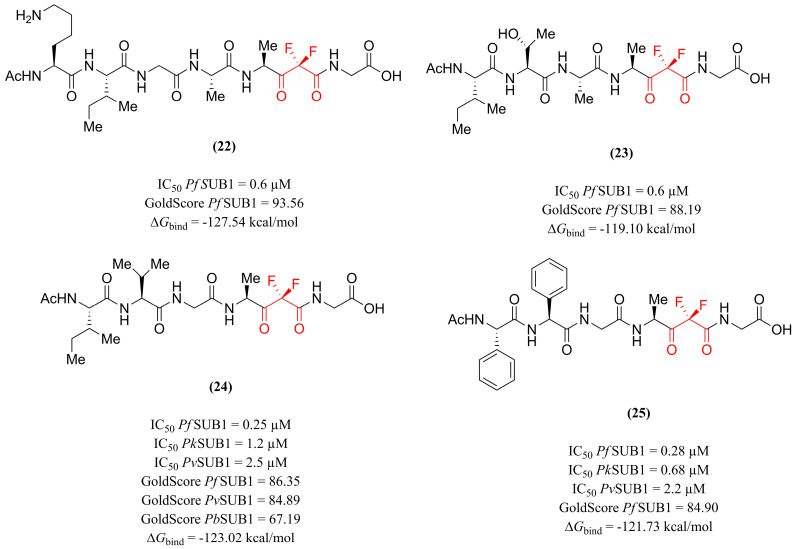
Chemical structures of peptidyl difluorostatones plasmodial SUB1 inhibitors.

Given this premise, Giovani et al. [122] reported the design, synthesis, and evaluation of difluorostatone-containing derivatives as inhibitors of *Pf*SUB1 protease. Using the structure and interactions formed by the substrate SERA4st1 as a model, the rational design approach consisted of replacing the cleavable peptide bond of the substrate with the electrophilic carbonyl group of difluorostatone. This group was strategically positioned due to its appropriate length, allowing it to project an *N*-terminal carboxylic group toward the K465 residue of the enzyme. Compounds **(22)** and **(23)** (Figure 8) exhibited high potency in the in vitro evaluation against *Pf*SUB1, with IC_50_ values of 0.6 µM. These structures differ by the absence of the *N*-terminal lysine in compound **(23)**, suggesting the feasibility of this molecular simplification. When evaluated in silico via molecular docking against *Pf*SUB1 (PDB ID: 4LVN), the compounds interacted with key residues (such as K465, S492, and H428) and presented GoldScore values of 93.56 and 88.19, respectively, both of which are higher than that of the substrate SERA4st1 (76.89). Additionally, MM/GBSA calculations indicated Δ*G*_bind_ values of −127.54 and −119.10 kcal/mol, respectively. Moreover, compounds **(22)** and **(23)** did not show activity against the mammalian proteases trypsin and chymotrypsin, indicating an interesting selectivity of these inhibitors for parasite proteases. These findings highlight the potential of difluorostatone-containing derivatives as promising *Pf*SUB1 inhibitors.

Subsequently, the compound’s series was expanded in the study conducted by Giovani et al. [123]. Extensive molecular modifications were carried out at different positions to determine the importance of each fragment to the pharmacological activity, including in the difluorostatone warhead. The investigation of the inhibitory potential of *Pf*SUB1 in vitro highlighted derivatives **(24)** and **(25)** (Figure 8) as the most promising (IC_50_ = 0.25 and 0.28 µM, respectively), emphasizing the feasibility of the molecular modifications performed for natural **(24)** and unnatural **(25)** amino acids on the P side of the molecule to increase affinity, in comparison to the previous study. In silico, the compounds presented Goldscores of 86.35 and 84.90, and Δ*G*_bind_ values of −123.02 and −121.73 kcal/mol, respectively. This study identified potent *Pf*SUB1 inhibitors, providing a more extensive understanding of the SAR of these compounds and highlighting the critical role of the difluorostatone warhead, as its absence resulted in a loss of biological activity.

Continuing the investigation with these compounds, Brogi et al. [23] proposed expanding the experimental evaluation and comprehensive computational investigation of the catalytic core of SUB1 to include six different *Plasmodium* species, using homology modeling and molecular docking approaches. Experimentally, the compounds were evaluated against *Pk*SUB1 and *Pv*SUB1, exhibiting low IC_50_ values in the micromolar range (1.2 and 2.5 µM for compound **(24)**, 0.68 and 2.2 µM for compound **(25)**). In the molecular docking evaluation, both compounds showed similar binding affinities, with derivative **(24)** standing out with Goldscore values of 84.89 (*Pv*SUB1) and 67.19 (*P. berghei* SUB1), indicating lower affinity in rodent *Pb*SUB1. This reduced interaction is consistent with structural differences, as a greater distance is observed between the electrophilic carbonyl and the catalytic residue Ser (5.0 Å in *Pf*SUB1 and 11.8 Å in *Pb*SUB1). Thus, these findings reaffirm the therapeutic potential of difluorostatone derivatives as promising candidates in the development of pan-specific antimalarial drugs.

#### 4.1.4. Peptidyl Boronic Acids

The boronic acid fragment is a versatile functional group found in drug structures of diverse origins and therapeutic purposes [124]. Its integration into peptide structures produces compounds capable of inhibiting enzymes via reversible covalent bonding, an interaction that is crucial for forming a stable and long-lasting complex. Accordingly, peptide boronic acids have been demonstrated to be potent inhibitors against serine proteases, such as subtilisin [125].

**Figure 9:**
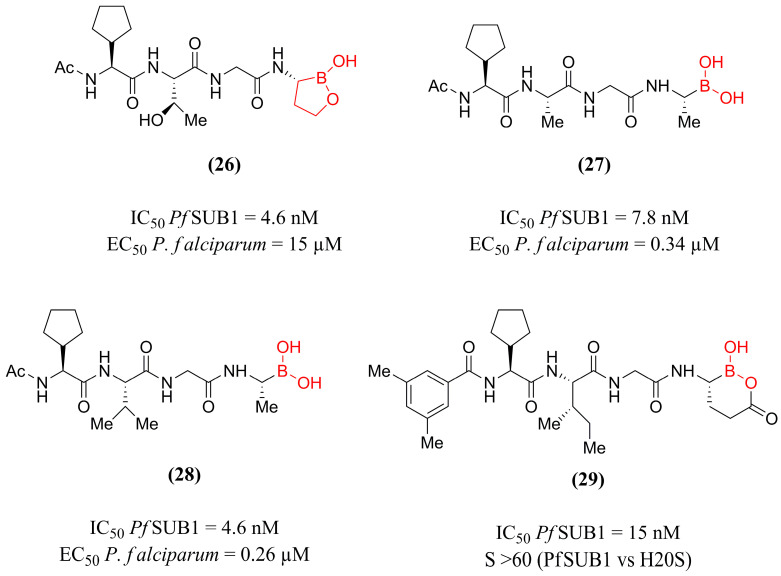
Chemical structures of peptidic boronic acid inhibitors of the protease *P. falciparum* SUB1.

Based on this premise, Lidumniece et al. [126] designed compounds based on the structure of previously published α-ketoamide peptide-derived inhibitors [18,120], aiming to improve the pharmacokinetics and potential of these compounds through the insertion of the boronic acid fragment. The experimental evaluation of *Pf*SUB inhibition demonstrated the potential of the 10 synthesized compounds, especially derivatives **(26)**, **(27)**, and **(28)** (Figure 9), which obtained IC_50_ values in the nanomolar range of 4.6, 7.8, and 4.6 nM, respectively.

The parasite growth inhibition of these compounds against *P. falciparum* strains complemented these results, reinforcing the efficacy of compounds **(27)** and **(28)** (EC_50_ = 0.34 and 0.26 µM, respectively). In contrast, compound **(26)** showed low cellular activity (EC_50_ = 15 µM), likely due to its low permeability. These findings established a favorable molecular modification pattern for the scaffold, wherein the substitution of the Thr group at the **P3** position with more hydrophobic fragments (Val, Ala) is beneficial for both pharmacokinetics and pharmacodynamics. Additionally, it is stated that the boronic acid moiety is crucial for binding to the catalytic serine and confers a dissociation half-life of approximately 31 min to compound **(28)**, consistent with the profile of a slowly reversible *Pf*SUB1 inhibitor. Additionally, when evaluated against mammalian trypsin-family serine proteases (trypsin, chymotrypsin, and elastase), derivative **(28)** demonstrated a high degree of selectivity for *Pf*SUB1. Ultimately, this study allowed the identification of highly potent compounds, encouraging further investigations with these promising derivatives.

Following this, the work developed by Withers-Martinez et al. [96] aimed to perform molecular modifications on the prototype **(28)** to obtain compounds with greater selectivity for *Pf*SUB1 over human proteasome (H20S). In this way, based on biological assays of fluorescence, cell viability, and computational modeling, compound **(29)** (Figure 9) emerged as the most promising candidate of this study. This derivative incorporates the boralactone group in its structure at position **P1** and showed enzymatic inhibition of *Pf*SUB1 at an IC_50_ of 15 nM, more than 60-fold selectivity for this enzyme over H20S (IC_50_ = 1000 nM), in contrast to the prototype **(28)**, which inhibits both targets with similar potency. The activity of the compound evaluated against strains B11 (wild-type) and IACS (*Pf*SUB1 knockdown) showed a 13x difference in sensitivity between strains, evidencing that compound **(29)** exerts its antimalarial growth-inhibitory activity through direct inhibition of *Pf*SUB1. Moreover, the selective inhibitor **(29)** exhibited a 27-fold reduction in HepG2 cell toxicity compared to the prototype **(28)**. Therefore, the authors point out boralactone-containing peptide inhibitors as new leads for antimalarial drug discovery.

### 4.2. Non-Peptide Structures

#### 4.2.1. Benzopyran Derivatives and Analogs

**Figure 10:**
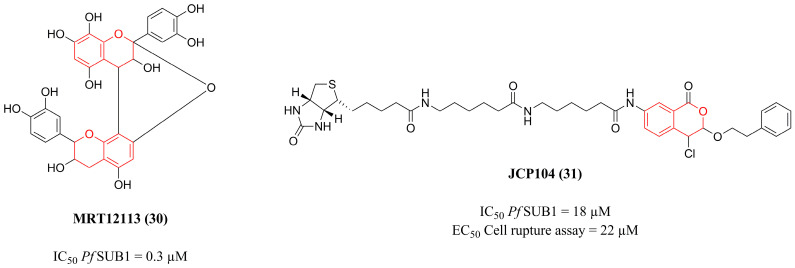
Benzopyran-based inhibitors of *P. falciparum* SUB1.

The study conducted by Yeoh et al. [127] involved a high-throughput screen of 170,000 low-molecular-weight compounds against recombinant PfSUB1, leading to the identification of the natural compound MRT12113 **(30)** (Figure 10) as a potent inhibitor (IC_50_ = 0.3 µM). In addition, the compound showed high selectivity to *Pf*SUB1 due to its lack of inhibition against other proteases up to a concentration of 50 µM, such as the related bacterial subtilisins BPN’ and Carlsberg; the mammalian serine proteases trypsin, chymotrypsin, and elastase; the cysteine protease papain; human caspase-3; the *P. falciparum* cysteine protease falcipain 2; the *P. falciparum* aspartic protease plasmepsin II; and the *P. falciparum* sheddase PfSUB2. Furthermore, the compound did not show toxicity against COS-7 cells or *Tetrahymena thermophila*, an organism related to the malaria parasite. When evaluated against *P. falciparum* culture, compound **(30)** inhibited host-cell rupture, and the few merozoites that were released displayed reduced competence for reinvasion, providing a great compound that can be explored in further work.

Further investigations performed by Koussis et al. [128] explored the mechanism of action of the compound mentioned before. In addition to preventing SERA maturation and blocking egress, as presented in the previous study, the compound demonstrated its ability to interfere in the ‘primary’ proteolytic cleavage of merozoite surface proteins (MSP1, MSP6, and MSP7) by complexing with *Pf*SUB1, resulting in the accumulation of the precursor forms of these proteins. This mechanism explains the positive impact on the decreased efficiency of merozoite invasion. These results provided insight into the role of *Pf*SUB1 in merozoite egress and reinvasion and identified a potent and selective inhibitor of this process.

Using a highly focused library of more than 1200 covalent serine and cysteine protease inhibitors, Arastu-Kapur et al. [129] sought to find compounds that block host-cell rupture by *P. falciparum*. Among these, JCP104 **(31)** (Figure 10), a biotinylated chloroisocoumarin-type inhibitor, was identified as the best serine protease inhibitor. It was observed that the relative potency of this compound in the rupture assay exactly correlates with its relative inhibitory effect on *Pf*SUB1, as compound **(31)** inhibits *Pf*SUB1 with an IC_50_ of 18 µM and acts by blocking cell rupture at an EC_50_ of 22 µM. Additionally, this compound blocked the maturation of serine repeat antigen protein 5 (SERA5), resulting in the accumulation of its precursors, P120 and P73. Therefore, the inhibition caused by the potent compound **(31)** on *Pf*SUB1 results in the blocking of *P. falciparum* cellular egress by preventing proteolytic processing in SERA5, representing an interesting scaffold for the development of antimalarial drugs.

#### 4.2.2. Quinoline Derivatives and Analogs

**Figure 11:**
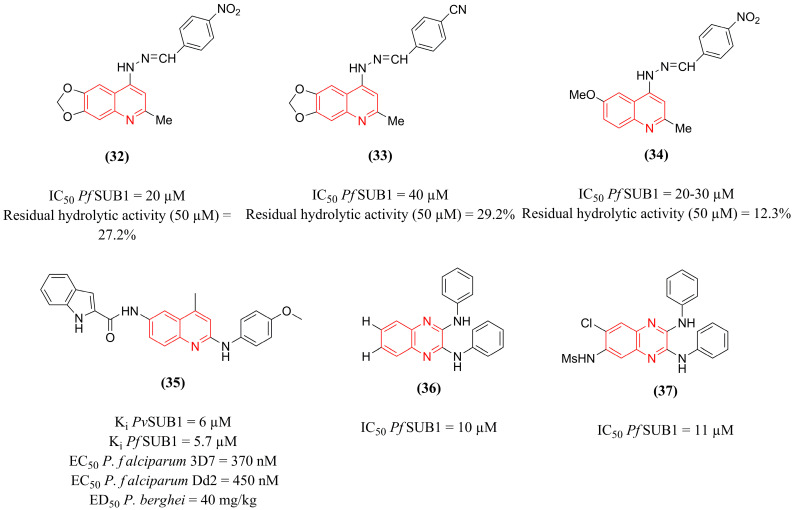
Structures of quinoline derivatives and analogs targeting plasmodial SUB1 protease.

In the search for small molecules with potential inhibition of *Pf*SUB1, Gemma et al. [130] performed high-throughput screening of approximately 450 peptide and non-peptide compounds. This approach led to the identification of a hit compound, quinolylhydrazone **(32)** (Figure 11), which presented an IC_50_ in *Pf*SUB1 of 20 µM and a residual hydrolytic activity percentage at 50 µM of 27.2%. Compound **(32)** was used as a prototype for the design and synthesis of analogs with structural modifications at the aromatic aryl substituent, the quinoline moiety, and the hydrazone linker, to establish a preliminary investigation of the structure-activity relationship between these compounds. Analog **(33)** (Figure 11), in which the nitro group of the prototype **(32)** is replaced with a cyano group, showed activity similar to that of the prototype, with a residual hydrolytic activity at 50 µM of 29.2% and a slightly reduced potency in enzyme inhibition (40 µM), which was still relevant. These findings are consistent with the electron-withdrawing capabilities of the substituents (NO_2_ > CN), indicating the importance of this feature in this portion of the structure and suggesting a safer substituent than the nitro group. In addition to this result, analog **(34)** (Figure 11), in which the dioxane ring fused to quinoline was replaced by the 6-methoxy group, showed an IC_50_ between 20 and 30 µM and a percentage of residual hydrolytic activity at 50 µM of 12.3%. Therefore, these compounds are established as essential lead structures for further development.

Combined with in silico techniques, the study by Bouillon et al. [110] included the construction of a three-dimensional model of the structures of *Pf*SUB1 and *Pv*SUB1 by homology modeling based on bacterial subtilisins. After obtaining these targets, a virtual screening was performed using approximately 150,000 compounds, resulting in the identification of 306 hits. Among these, 37 compounds were evaluated in vitro against *Pv*SUB1 and *Pf*SUB1, with quinolinic compound **(35)** (Figure 11) standing out, exhibiting K_i_ values of 6 and 5.7 µM, respectively. When assessed against parasitic strains of *P. falciparum*, this compound showed potent antiplasmodial activity, with an EC_50_ of 370 and 450 nM on the chloroquine-sensitive 3D7 clone and chloroquine-resistant Dd2 clone, respectively. Additionally, compound **(35)** was evaluated in vivo against *P. berghei* blood stages and presented an estimated ED_50_ value of 40 mg/kg, with no signs of toxicity observed. In conclusion, compound **(35)** inhibited endogenous *Pf*SUB1 by blocking the maturation of its natural substrate *Pf*SERA5, inhibiting parasite evasion and erythrocyte invasion.

2,3-Bis(phenylamino)quinoxalines have been identified as a novel class of malarial protease PfSUB1 inhibitors, as reported by Kher et al. [131]. When screening the Malaria Box, a collection of 400 compounds with confirmed activity against the blood stage of *P. falciparum*, compound **(36)** (Figure 11) was identified as a *Pf*SUB1 inhibitor (IC_50_ = 10 µM). Subsequently, 17 analogs were synthesized, among which compound **(37)** (IC_50_ = 11 µM) (Figure 11) stood out for presenting *Pf*SUB1 inhibitory activity comparable to prototype **(36)**, with substitutions in positions R_1_ (Cl) and R_2_ (MsNH) of the quinoxaline ring. The other analogs led to a decrease or loss of inhibitory potential. Nevertheless, the preliminary SAR provided important information for the development of novel and improved inhibitors.

#### 4.2.3. Triterpene Derivatives

**Figure 12:**
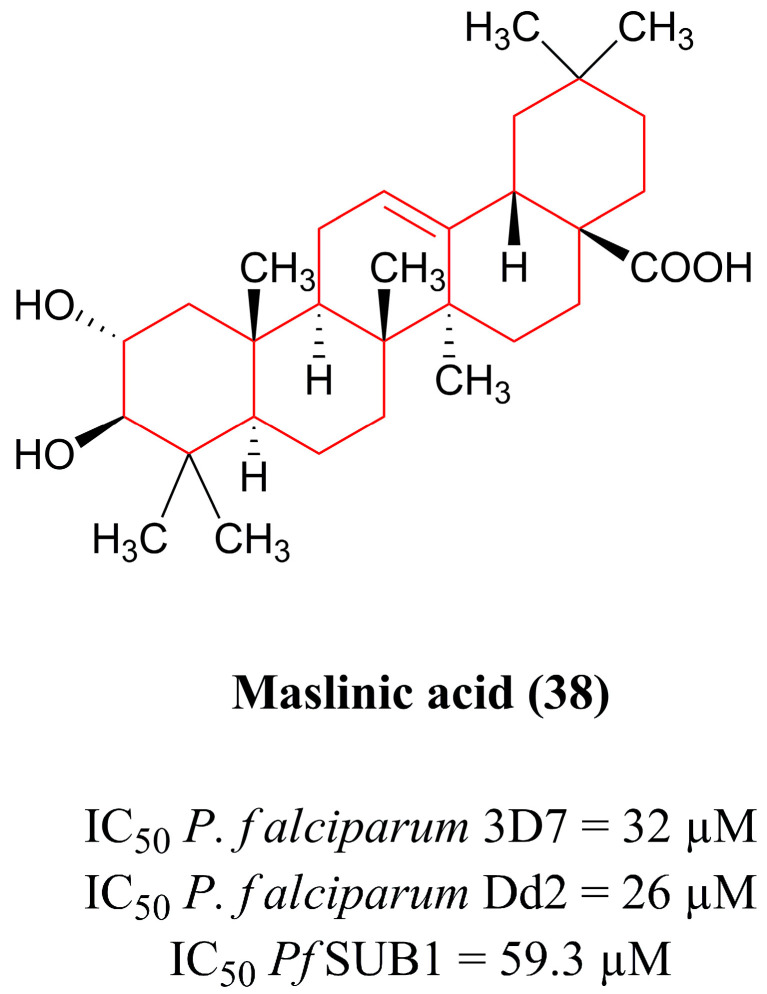
Chemical structure of triterpenoid compound with SUB1 protease inhibitory activity.

Maslinic acid **(38)** (Figure 12) is a natural pentacyclic triterpene with a broad spectrum of reported therapeutic activities. It exhibits antiplasmodial activity against chloroquine-sensitive (3D7) and chloroquine-resistant (Dd2) strains of *P. falciparum*, with IC_50_ values of 32 and 26 μM, respectively. Its antimalarial effect has been reported in multiple intraerythrocytic stages, and, depending on the dose and incubation time, it behaves as a plasmodial parasitostatic compound, displaying accumulation of ring, trophozoite, or schizont intraerythrocytic forms [132]. Additionally, this triterpene demonstrated in vivo activity in the *P. yoelii* 17XL murine model. ICR mice treated with compound **(38)** increased the survival rate from 20% to 80%, showing an arrest of parasite maturation from day 3 to 7 after infection, leading to synchronization of the intraerythrocytic cycle and accumulation of schizonts by day 6, proving that this compound also behaves as a parasitostatic agent in vivo [133].

In view of this, Moneriz et al. [134] investigated the possible mechanisms underlying the antiplasmodial effect of compound **(38)** by evaluating it against different proteases, including *P. falciparum* subtilisin. The in vitro assay of enzyme inhibition demonstrated that **(38)** presents an IC_50_ of 59.3 μM against *Pf*SUB1, resulting in a blockade of MSP1 processing in *P. falciparum* cultures at the schizont stage. These results indicate that at least *Pf*SUB1 is inhibited by **(38)** in erythrocyte cultures, which may contribute to the arrest of the *P. falciparum* infective cycle detected in treated cultures, corroborating that this compound behaves as a serine protease inhibitor.

## 5. Challenges and Opportunities to Discover Antimalarial Drugs Targeting Subtilisins

Malaria continues to be a significant public health problem, and there is an urgent need to discover new threats to this pathogen. Therefore, medicinal chemistry studies are necessary to improve approaches and provide new, safe drugs. Furthermore, identifying new targets is crucial, especially in overcoming the parasite’s resistance to existing treatments. In this way, subtilisin provides an opportunity in drug discovery. Therefore, one of the significant challenges to be overcome in the discovery of subtilisin inhibitors is the current limited number of crystallographic structures available for this target. Therefore, for rational design, primarily through CADD approaches, such as SBDD, the availability of more X-ray structures of the target, particularly for SUB3, is essential. This generates critical new information and the possibility of discovering new inhibitors [117].

Furthermore, the complete elucidation of the catalysis and substrate binding mechanisms still needs to be fully elucidated. Thus, in addition to X-ray structures, biochemical studies focusing on catalysis are essential for understanding the binding modes and, consequently, the structural characteristics necessary for designing a promising inhibitor of SUB1 and especially SUB2 and SUB3 [117].

To date, few classes of chemical scaffolds are known to inhibit subtilisin. In particular, there is a need for more inhibitors that target SUB2 and SUB3, thereby identifying whether their inhibition contributes to slowing disease progression. Thus, there are numerous opportunities for drug design to evaluate new chemical scaffolds and identify classes of compounds that may be effective subtilisin inhibitors, thereby generating promising antimalarial drugs [117,135].

Ultimately, it is evident that the development of new antimalarial drugs necessitates overcoming the parasite’s mechanisms of drug resistance. Thus, subtilisin inhibitors may provide a novel mechanism of action that can overcome resistance mechanisms. Therefore, the importance of developing new inhibitors as a focus for subtilisin inhibition to demonstrate its action against resistant strains of *Plasmodium* sp. is notable, opening new opportunities in drug discovery [117].

## 6. Conclusions and Future Outlook

Malaria, an ancient disease, continues to raise concerns among health agencies and threatens the population living in tropical countries. Despite the existence of treatments, a need remains for new, safer, and more effective drugs that can overcome the parasite’s resistance mechanisms. Thus, subtilisin inhibition offers an excellent mechanism of action that can be explored by researchers worldwide in the development of new antimalarials. As shown here, the primary inhibitors are peptide analogs, with promising results that encourage further optimization studies. On the other hand, non-peptide analogs, such as quinoline derivatives, benzopyran, and triterpene, demonstrate their importance and can be explored in future optimization studies. Indeed, it is still necessary to explore new chemical scaffolds with increased affinity against these enzymes, providing key information for the development of antimalarial drugs. Furthermore, exploring SUB1, it is clear that CADD studies in SBDD approaches suggest that high-affinity inhibitors have interactions with the triad catalytic Asp^372^, His^428^, and Ser^606^, but interactions with Leu^469^ and Gly^467^, and especially with Asn^520^, demonstrate their importance and can yield excellent results in optimization studies and the discovery of new antimalarials. We hope that our study will provide new horizons for researchers worldwide in the search for a new, safer, more effective, resistance-free, and innovative antimalarial drug.

## Figures and Tables

**Table 2:** Comparative analysis of catalytic mechanism features in *Pf*SUB1, *Pf*SUB2, and *Pf*SUB3 proteases from *Plasmodium falciparum*.

Enzyme	Structural Data	S1 Binding Pocket	S2 Pocket Feature	S4 Pocket Characteristic	Oxyanion Hole Residue
*Pf*SUB1	Crystal structure	Polar; favors acidic P1 via Ser^490^, Ser^517^, Ser^519^	Constrained by Lys^465^; small	Hydrophobic (Phe^491^, Phe^493^, Phe^500^)	Asn^520^; redox-controlled loop
*Pf*SUB2	No structure	Likely broad/hydrophobic	Likely flexible for large substrates	Presumed broader hydrophobic	Roughly canonical
*Pf*SUB3	No structure	Unknown; predicted unique phenylalanine may alter shape	Unknown	Undetermined; speculative	Canonical based on homology

## Data Availability

Not applicable.
